# Assessment of the European Union's illicit trade agreements with the four major Transnational Tobacco Companies

**DOI:** 10.1136/tobaccocontrol-2014-052218

**Published:** 2015-05-28

**Authors:** Luk Joossens, Anna B Gilmore, Michal Stoklosa, Hana Ross

**Affiliations:** 1 Association of the European Cancer Leagues & Tobacco Control Expert, Foundation Against Cancer, Brussels, Belgium; 2 Department for Health, University of Bath and UK Centre for Tobacco and Alcohol Studies, Bath, UK; 3 Economic and Health Policy Research, American Cancer Society, Atlanta, USA; 4 University of Cape Town, Cape Town, South Africa

**Keywords:** Illegal tobacco products, Tobacco industry, Advocacy

## Abstract

To address the illicit cigarette trade, the European Union (EU) has signed agreements with the four major Transnational Tobacco Companies (TTCs) that involve establishing extensive systems of cooperation. All agreements foresee two types of payments: annual payments (totalling US$ 1.9 billion over 20 years) and supplementary seizure payments, equivalent to 100% of the evaded taxes in the event of seizures of their products. While limited by the fundamental lack of transparency in this area, our analysis suggests that these agreements have served largely to secure the TTCs’ interests and are threatening progress in tobacco control. The seizure payments are paltry and a wholly inadequate deterrent to TTC involvement in illicit trade. Despite the agreements, growing evidence indicates the TTCs remain involved in the illicit trade or are at best failing to secure their supply chains as required by the agreements. The intention of the seizure-based payments to deter the tobacco industry from further involvement in the illicit cigarette trade has failed because the agreements contain too many loopholes that provide TTCs with both the incentive and opportunity to classify seized cigarettes as counterfeit. In addition, the shifting nature of cigarette smuggling from larger to smaller consignments often results in seizures that are too small to qualify for the payments. Consequently, the seizure payments represent a tiny fraction of the revenue lost from cigarette smuggling, between 2004 and 2012, 0.08% of the estimated losses due to illicit cigarette trade in the EU. Our evidence suggests the EU should end these agreements.

## Introduction

A key element of the European Union's (EU) policy to combat the illicit cigarette trade is its collaboration with the tobacco industry.^1^ The EU has signed illicit trade agreements with the four major Transnational Tobacco Companies (TTCs): in 2004 with Philip Morris (PM), which includes Philip Morris International (PMI); in 2007 with Japan Tobacco International (JTI); and in 2010 with British American Tobacco (BAT) and Imperial Tobacco Limited (ITL).^2–5^ At least three of the four agreements were signed to settle or avoid legal disputes between the companies and the EU in relation to involvement of those companies in cigarette smuggling. All established extensive systems of cooperation between the TTCs and the EU at a time when the broader regulatory trend was one of exclusion.

Since the Agreement with PMI ends in July 2016, negotiations to explore a possible extension are currently underway.^6^ This article therefore aims to inform those negotiations by examining the effectiveness of these agreements. It begins by outlining the background to the agreements including the history of TTC involvement in the illicit cigarette trade globally and in the EU, and the development and nature of these agreements. The aim of this paper is to critically assess the agreements and their execution in the EU. We also discuss the implications of these agreements for tobacco control.

## The history of the tobacco industry's involvement in the illicit tobacco trade

Tobacco companies make their profits when they sell to traders, regardless of whether the cigarettes are then sold legally or illegally.^7^ ^8^ Evidence of the direct and indirect involvement of the tobacco industry in cigarette smuggling is well documented—in internal documents that TTCs were forced to release in the course of litigation,^9–14^ their own admission^15^ and court judgements.^16^ Since 1997, there have been several official investigations and subsequent court cases in different parts of the world (Hong Kong, Canada, Colombia) that have accused the industry of supplying the smuggled cigarettes or at least of being aware of the illegal destination of their products.^17–22^

In the 1990s, American cigarette brands were a key element of the contraband trade in the EU.^23^ ^24^ An EU investigation on smuggling activities started in 1998. It culminated on 3 November 2000, when the EU filed a lawsuit in the US District Court in New York, against PM, RJ Reynolds and Japan Tobacco, which had by then acquired the international division of RJ Reynolds, alleging that these tobacco companies were guilty, in effect, of controlling entire smuggling operations and accusing the companies of “an ongoing global scheme to smuggle cigarettes, launder the proceeds of narcotics trafficking, obstruct government oversight of the tobacco industry, fix prices, bribe foreign public officials and conduct illegal trade with terrorist groups and state sponsors of terrorism”.^25^ In 2000 and 2001, 10 EU countries, led by Italy, joined the lawsuit.^26^

Additional allegations were filed by the EU and ten EU governments against RJ Reynolds in the same court in October 2002 on the company's engagement in organised crime, money laundering and narcotics trafficking and in transactions that financed both the Iraqi regime under Saddam Hussein and terrorist groups.^27^ ^28^ While this second lawsuit against RJ Reynolds is still ongoing,^29^ PM and JTI settled their smuggling disputes with the EU in 2004 and 2007, respectively.^4^

## Agreements with the four major tobacco companies

In parallel with the legal proceedings, confidential discussions began in late 2001 between the European Commission and PM on a possible agreement to cooperate in combating illicit cigarettes.^4^ In 2004, the EU and 10 Member States dropped the case against PM in exchange for an enforceable and legally binding agreement. A similar agreement settling a legal dispute was concluded with JTI in December 2007. Two additional agreements were signed with BAT in July 2010, and ITL in September 2010. An overview of the main characteristics of the four agreements is presented in table 1.

**Table 1:** Characteristics of the agreements between the TTC's and the EU

Company	Total of annual payments US$	Date of signature	End	Settlement and/or discharge of legal claims	Renewal clause	Termination clause
PM	1 billion	9/7/2004	9/7/2016	Yes	Yes	No
JTI	400 million	14/12/2007	14/12/2022	Yes	No	Yes
BAT	200 million	15/7/2010	15/7/2030	Unknown (see text)	Yes	Yes
ITL	300 million	27/9/2010	27/9/2030	Yes	Yes	Yes

Source: OLAF.^5^

BAT, British American Tobacco; EU, European Union; JTI, Japan Tobacco International; ITL, Imperial Tobacco Limited; PM, Philip Morris; TTC, Transnational Tobacco Companies.

Although the agreements with BAT and ITL were not part of a legal settlement, ITL's agreement releases the company from future liability for smuggling, and it is possible that the BAT agreement may serve the same purpose. Alongside the main agreement, ITL signed a parallel agreement (called Mutual Cessation Agreement) that “absolutely, unconditionally and irrevocably fully release and discharge ITL Group Companies and their successors, Agents and Assigns from any and all EU Claims”.^5^ A press release from Her Majesty's Revenue and Customs in the UK suggests the BAT agreement has a similar clause noting that “the manufacturers (BAT) are released from any civil claims arising out of past conduct relating to illicit trade”.^30^ However, this information is not part of the publicly available BAT-EU agreement.^5^ Three of the four agreements (except with PM) also give TTCs the right to terminate the monetary payments if the agreements fail to meet their “reasonable expectations” of benefit.^5^

The four agreements foresee two types of payments to the European Commission and the Member States: annual fixed payments (see table 1) and supplementary seizure-based payments (see table 2). The fixed payments total 1.9 billion US dollars paid in annuities from 2004 to 2030. PM pays the European Commission and Member States and its lawyers a total of $1.250 billion in annual payments (payment of $1 billion over 12 years and $250 million legal fees). JTI's total of annual payments are $400 million over 15 years, BAT's total of annual payments are $200 million over 20 years, and ITL's annual payments are $300 million over 20 years. These payments are generally considered compensation for the losses incurred through the TTC's smuggling activities. For instance, Italy—a prominent victim of PM smuggling activities—received the largest proportion (28.62% of the 1 billion paid by PM).^31^

**Table 2:** Cigarette seizure payments in the EU as a result of agreements with four major tobacco companies

Year	Seizure payments (EURO)
2006	20 289 472
2007	10 342 796
2008	6 922 838
2009	11 178 975
2010	7 754 716
2011	10 098 035
2012	4 141 791
2006–2012	70 728 624

Source: Anna Gilmore—documents obtained via access to documents legislation.^38^

EU, European Union.

In addition to the annual payments outlined above, a key feature of the agreements is the specification of seizure payments if TTCs fail to control their supply of cigarettes to the illegal market. The companies agreed to make payments equivalent to 100% of the evaded taxes in the event of any seizures of their genuine products above 50.000 cigarettes in the EU countries that were party to the Agreement. If seizures of their genuine products in the Member States during a year exceed the baseline amounts defined in the Agreements (originally set at 90 million cigarettes in the PM agreement, 90 million cigarettes in the JTI and ITL agreements and 150 million cigarettes in the BAT agreement), the tobacco companies must pay 500% of the evaded duties and taxes.^5^ Although these baseline amounts have not yet been reached, the PMI baseline was raised from 90 million cigarettes to 450 million in 2011,^32^ substantially reducing the likelihood of PMI, spun off from PM in 2008, ever paying the 500% penalty.

Some countries earmark the payments to fight illicit cigarette trade, while others direct the funds to the general budget.^2^ All agreements also required TTCs to secure the supply chain through a range of measures, including product marking and tracking and tracing provisions. The agreements are enforceable, but all arbitral proceedings are kept confidential.^5^

## Assessment of the agreements

Full evaluation of the agreements is almost impossible, as there are no independent publicly available data on the origins and brands of illicit tobacco products and the size of the illicit market in the EU (personal communication, Corneliu Hoedlmayr, International Relations Officer, OLAF, 6 May 2014). The only publicly available data on the EU illicit cigarette trade over time are data produced by KPMG for the tobacco industry.^33^ The KPMG data have been reviewed elsewhere and although the underlying methodology was commended, significant concerns were raised about the accuracy of the data and the extent to which they serve the TTCs’ interests.^34^ Relying on industry data is therefore highly problematic. We, therefore, limit our analysis to examining the agreements and their possible impact on tobacco control, and thus on public health.

## Findings

### Lack of transparency

A key concern is the lack of transparency surrounding the negotiations for, and ongoing management of, the agreements and illicit trade more generally. Also, parts of the agreements are not public. For example, parts of the publically available text of the agreement with ILT have been “omitted due to a request for confidential treatment”.^35^ Access to document requests reveal that the correspondence between the European Anti-Fraud Office (OLAF) and the four tobacco manufacturers amounts to tens of thousands of documents, and that OLAF has attended numerous meetings with PMI, JTI, BAT and ITL as part of the agreements.^36^ Yet, not only is there no comprehensive list of these documents,^36^ but requests for these documents, and related information through parliamentary questions and access to documents requests, are often refused on the basis of confidentiality.^36–38^ For example, a parliamentary question could not obtain information on the seizure payments for one of the important contraband brands, Classic, an ITL brand,^39^ which was in 2008 the third most seized cigarette brand in the EU.^40^ The European Commission stated that no information could be provided due to “the rules governing the treatment of confidential information (…)”.^39^ A freedom of information request on the PMI tracking and tracing system, Codentify, revealed that between January 2008 and November 2011, there were 17 documents of correspondence between OLAF and PMI on this subject. Ten documents were not made public because their disclosure could harm the interests of the Commission during the FCTC negotiations, and “OLAF's relations with companies potentially involved in the possible implementation of tracking and tracing systems”.^37^ It remains unclear how the release of documents in 2012 could harm the Commission's negotiating position as the consensus on tracking and tracing systems through the WHO FCTC's Illicit Trade Protocol was reached in March 2010.^41^ Further, this consensus was that the control of tracking and tracing should remain under control of governments and not be delegated to the tobacco industry (§2 and §10 of the article 8 of the Illicit trade Protocol), raising serious concerns about the Commission's response.^42^

### Inadequate deterrent

The seizure-based payments are the agreements’ main mechanism for deterring the TTCs from further involvement in the illicit cigarette trade by punishing them each time there is a large seizure of their cigarettes. This should also allow EU Member States to recover the taxes lost. Cigarettes are highly taxed products and OLAF estimates that, on average, a container with 10 million cigarettes represents 2 million EURO of lost tax revenue.^43^

Information on seizure payments is not made public but was obtained via access to documents legislation (table 2).^38^ In the period 2004–2012, a total of €70,728,624 (around US$ 100 million) in seizure payments was made by PMI, JTI and ITL, or, on average, €8.3 million annually. BAT has made no seizure payments to date.^38^ In 2012, only €4.1 million was paid in seizure payments.^38^ This means that the seizure payments were made for approximately 20 million seized cigarettes,^43^ just 0.5% of the 3.8 billion cigarettes seized in the EU in 2012.^2^

These payments represent a tiny fraction of the revenue lost from cigarette smuggling. Based on seizure data in the period 2005–2011, OLAF estimates the financial losses due to illicit cigarette trade at €10 billion annually in the EU.^1^ The €8.3 million average annual seizure payments are only 0.08% of those estimated losses.

There are two main reasons these seizure payments are so small. First, only large seizures qualify for payments, and since the agreements were reached, the modus operandi of cigarette smuggling in Europe has changed. While very large consignments of illicit cigarettes dominated at the time when the agreements were negotiated, most illicit cigarette seizures now consist of substantially smaller consignments. This trend has been confirmed by the World Customs Organization in its 2013 illicit trade report.^44^ In Poland, the country with the highest level of cigarette seizures in the EU, the average seizure in 2011 was around 5200 cigarettes, and for certain brands, such as BAT's Viceroy, the average seizure amount was even lower: 1615 cigarettes.^45^ This means that the threshold of 50 000 cigarettes needed to recover taxes and duties lost has become increasingly difficult to meet. The shifting nature of illicit trade has been even recognised by BAT when it agreed to a lower qualifying threshold of 7500 cigarettes in its 1 August 2014 agreement.^46^ However, this new threshold still remains higher than, for instance, the average seizure in Poland, and is therefore not likely to be effective.

Second, payments only apply to genuine TTC cigarettes and not counterfeits, and yet customs officials rely on the industry to determine whether cigarettes are counterfeit (not eligible for seizure-based payments) or genuine (eligible for the payments). This provides a motivation and opportunity for TTCs to claim that the seized cigarettes are counterfeit. According to the agreements, the relevant manufacturer is entitled to examine the seized cigarettes, and send a report to OLAF. If the manufacturer concludes that the cigarettes are counterfeit, the report must contain documentation and examination results demonstrating that conclusion.^4^ If OLAF, or any participating Member State, disagree with the conclusion that the seized cigarettes are counterfeit, the matter is referred to an independent laboratory, designated by mutual agreement of the parties, for final determination.^4^ Since the first agreement came into effect, until 31 October 2013, the seized cigarettes have never been analysed by an independent laboratory and all determination has instead been based on examinations by the TTCs.^47^ During this period, Member States submitted a total of 6,261 seizure notices (for seizures of more than 50 000 cigarettes) under the agreements. Out of the total number of seized cigarettes, 3.2 billion (78%) were claimed to be counterfeit cigarettes.^47^ Among seized cigarettes of PMI's brands, PMI claimed that 92% of them were counterfeit cigarettes in 2011.^48^ Yet this very high level of counterfeit cigarettes among seizures is inconsistent with the industry's own estimates of counterfeits on the illicit market. PMI's estimate for the illicit cigarette market globally is that only 1% was counterfeit in 2012.^49^ For the EU market, a PMI-commissioned study states that 16% of illicit PM cigarettes consumed in the EU were counterfeit in 2011.^48^ Thus, the industry-estimated prevalence of counterfeits among seized cigarettes is almost six times higher than the prevalence of counterfeits among consumed cigarettes. This would imply that seizure data are unrepresentative of the nature of the illicit market. There are two potential reasons for this. One could be that large seizures are more likely to contain counterfeit than genuine TTC products (and therefore under-represent the real nature of the illicit market in Europe). The other is that TTCs are classifying too many illicit cigarettes as counterfeit in large seizures. Either way, it is clear that the seizure payments do not reflect the size of the illicit market attributable to the TTCs’ genuine products.

### The industry's tracking and tracing system

Obliged by the agreements to implement a tracking and tracing system, the TTCs developed their own system, named Codentify. The effectiveness of this system has previously been assessed. It has been found that the industry's system does not meet requirements outlined in Article 8 of the FCTC's protocol on illicit trade,^50^ which defines that the tracking and tracing system must be “controlled by the Party”.^51^ In addition, the industry-managed tracking and tracing obligation has recently lost its significance due to an EU tracking and tracing system established in Articles 15 and 16 of the Tobacco Products Directive 2014/40/EU of 3 April 2014.^52^ The new Directive introduces an EU-wide tracking and tracing system for the legal supply chain, and visible and invisible security features (eg, holograms) that should facilitate law enforcement, and help authorities and consumers detect illicit tobacco products.

### Has TTC involvement in illicit cigarette trade declined or ceased since the agreements between the EU and the TTCs were reached?

The intention of the agreements was to crack down on smuggling and put in place what it called a “zero tolerance policy” toward illicit shipments.^53^ Yet several reports indicate that the TTC's have remained involved in the trade since their deals were reached. Investigative journalists obtained internal JTI records from whistle-blowers indicating that the company remained involved in the illicit trade and had been less than compliant with the EU agreement.^53^ ^54^ OLAF has been officially investigating this case since December 2011,^55^ but has yet to make a public statement or come to a conclusion. Other sources point to the TTC's continued complicity in cigarette smuggling to and through Bulgaria between 2000 and 2010, again, after its agreement was reached.^56^ PMI data in 2011 indicate that 21% of all illicit cigarettes in the EU are PMI's own genuine brands.^48^ At best, this indicates PMI's failure to secure its supply chain as the agreement envisaged. Concerns have been raised about the cigarette brand Classic, produced by Imperial Tobacco, Ukraine, and one of the most seized cigarette brands in the EU in 2008,^40^ particularly in light of evidence that the TTCs have been over-producing cigarettes in Ukraine in the knowledge they would enter the illicit market.^57^ In 2014, BAT was fined £650 000 ($1 m; €820 k) by UK tax authorities for oversupplying its products to Belgium.^58^

### What impact has the collaborative approach outlined in the agreements had on tobacco control more broadly?

A key element of the agreements is that they establish extensive systems of cooperation between the manufacturers, the EU and Member States, an element of the agreements that TTCs have heavily emphasised.^59^ ^60^ It is noteworthy that the agreements were reached at a time the TTCs were increasingly being excluded from the policy arena via article 5.3 of the FCTC, which was adopted in 2003 with Guidelines for Article 5.3 agreed in November 2008.^61^ Illicit tobacco provided a perfect opportunity for the TTCs, despite their inauspicious history, to signal shared concerns with policy makers and convince authorities that they were acceptable partners in addressing a trade in which they had previously been complicit.^62^ The potential danger of this collaborative approach threatening tobacco control if norms of collaboration in illicit reached into other areas of tobacco control has previously been raised.^62^ Those threats have been particularly apparent during the revision of the EU Tobacco Products Directive.^63^ ^64^ Michel Petite, the Director-General of the European Commission's legal service at the time the first two agreements were negotiated, who played a key role in such efforts, now works for PMI in his new position at legal firm Clifford Chance.^63^ The normalisation of relationships between the TTCs and EU public officials is further illustrated by the nomination of Michel Petite as chair of the EU Commission's Ad hoc Ethical Committee in 2009 and 2012.^65^ The close relationship between OLAF and the TTCs, indicated through the documents outlined above, raises questions, given the concerns about OLAF's role in the scandal surrounding the resignation of Health Commissioner John Dalli,^66^ ^67^ the lack of progress of OLAF's investigation into JTI's alleged ongoing involvement in illicit trade,^55^ and OLAF's apparent willingness to involve TTCs in the implementation of tracking and tracing systems under the FCTC's Illicit Trade Protocol, contrary to the protocol itself.^37^

A further concern is that the TTCs appear to have used the agreements with the EU, which are heavily promoted on their websites,^68^ ^69^ to negotiate an increasing number of similar agreements nationally and internationally.^70^ In 2011, PMI and INTERPOL, the world's largest police organisation, agreed on a deal in which PMI donated €15 million to fund a global initiative against illicit goods trafficking,^71^ which would promote PMI's Codentify system.^50^

## Discussion

While limited by the fundamental lack of transparency in this area, our analysis suggests that the agreements have served largely to secure the TTCs interests, reinforced cooperation between the manufacturers, the EU and Member States, and are threatening progress in tobacco control. The seizure payments are paltry and are a wholly inadequate deterrent to TTC involvement in illicit trade. The intention of the seizure-based payments to deter the tobacco industry from further involvement in the illicit cigarette trade has failed because the agreements contain too many loopholes. The industry has both the incentive and opportunity to classify seized cigarettes as counterfeit; and, despite the nature of cigarette smuggling having changed from large to small consignments, seizure payments are due only on large consignments. Further, even the tobacco industry's own data suggest that seizure data (which are based only on large seizures) significantly under-represent the proportion of genuine TTC brands in the illicit market. Consequently, the payments provide no incentives to stop cigarette smuggling and the recovered value of taxes is minimal.

Growing evidence indicates that, despite the agreements, the TTCs remain involved in the illicit trade or are, at best, failing to secure their supply chains as required by the agreements. The agreements instead appear to be part of the TTCs’ strategy to establish alliances and partnerships with authorities at the national and international levels to position the tobacco industry as part of the solution to the illicit tobacco trade. By establishing extensive yet opaque collaboration between the TTCs and the Commission, the agreements threaten tobacco control within the EU. By enabling the industry to promote the agreements as an effective model of collaboration, they are also threatening tobacco control internationally. For this reason, many parties to the FCTC were opposed to the inclusion of “legally binding and enforceable agreements” in the text of the draft protocol during the last round of the negotiations of the illicit trade protocol in 2012.^72^

The agreements are also not in line with Article 5.3 of the WHO's Framework Convention on Tobacco Control (FCTC), which all 28 EU countries and the EU have ratified, requiring that “in setting and implementing their public health policies with respect to tobacco control, parties shall act to protect these policies from commercial and other vested interests of the tobacco industry in accordance with national law”. The EU's contradictory approach to Article 5.3 and industry collaboration is illustrated by its opinion, expressed at the Fifth and Sixth Conference of the Parties to the FCTC in November 2012^73^ and October 2014,^74^ that INTERPOL should not to be granted an observer status since it received funding from the tobacco industry. Yet a similar relationship exists between the EU and the tobacco industry: with no linked legal action to prompt a deal, the EU accepted $500 million (€400 million) from BAT and ITL, 26 times larger than PMI's donation to INTERPOL.

In three of the four agreements, the tobacco companies have the right to terminate the monetary payments if there are significant failures of their “reasonable expectations” as to their benefits under the agreements.^3^ No definition of “reasonable expectations” is given publicly and an independent legal opinion notes that “because these expectations are to be assessed by reference to documents, correspondence and agreements which are not publicly available, the breadth of the circumstances in which the EU and Member States might risk termination by the companies cannot be determined.”^3^ We believe that there are significant failures of “reasonable expectations” of the public health community with respect to these agreements.

The tracking and tracing obligations in the agreements lost their significance when the EU adopted its Tobacco Products Directive 2014/40/EU, with an EU-wide tracking and tracing system foreseen in Articles 15 and 16.

The EU has already begun its negotiations with PMI to possibly extend its 2004 agreement, while at the same time PMI is challenging the 2014 Tobacco Products Directive in the EU Court of Justice.^75^ We conclude that the agreements have little or no added value and conflict with the article 5.3 of the WHO FCTC. The EU was correct when it expressed a disapproval about the relationship between INTERPOL and PMI and confirmed publicly that “the interests of the tobacco industry are fundamentally opposed to public health”.^73^ It should apply the same standard to its own deal with TTCs. Our evidence suggests the EU should not extend its agreement with PMI and, if legally possible, should end the agreements with the other three tobacco companies.

**What this paper adds**

- The involvement of the tobacco industry in cigarette smuggling, both direct and indirect, has been well documented. Governments have addressed this type of tax evasion in different ways, including by signing agreements with the tobacco industry to control their supply chain. The best known example of such arrangements is between the four major Transnational Tobacco Companies (TTCs) and the European Union (EU). However, there is no comprehensive assessment of the impact of such agreements in the scientific literature, while the tobacco industry continues to promote such agreements globally.
- We used multiple sources to evaluate the agreements between the EU Commission, EU Member States and major tobacco companies. Despite the lack of data and the secrecy surrounding these contracts, the evidence suggests that they are ineffective. Seizure payments, for instance, provide no incentive for the TTCs to end their involvement in cigarette smuggling because the agreements contain too many loopholes and the recovered value of taxes is minimal compared to the financial losses due to illicit cigarette trade.
